# Association of early blood glucose metrics with short- and long-term prognosis in acute myocardial infarction patients: a retrospective cohort study

**DOI:** 10.1186/s12872-026-05842-5

**Published:** 2026-04-08

**Authors:** Ji Jia, Si-ming Tao, Su-li Bao, Ying-bo Shuang, Ke-xin Peng, Yi-hua Luo, Hua Jiang, Ai-ni Yang, Hua-lei Dai, Zhi-gang Yang, Wei Wei, Yun-zhu Peng

**Affiliations:** 1 Department of Cardiology, The Affiliated Hospital of Yunnan University, No. 176, Qingnian Street, Wuhua District, Kunming, Yunnan 650021 China; 2 Kunming Medical University, Kunming, Yunnan 650021 China; 3 Yunnan University, Kunming, Yunnan 650021 China

**Keywords:** Acute myocardial infarction, Blood glucose variability, Stress hyperglycemia ratio, Admission glucose, Prognosis, Mortality, MIMIC-IV

## Abstract

**Background:**

Acute myocardial infarction (AMI) remains a major cause of morbidity and mortality globally. Recent evidence suggests that beyond single blood glucose measurements, early glycemic fluctuations and stress hyperglycemia may have significant prognostic implications in AMI patients. This study investigates the association between early blood glucose variability indices—including glucose standard deviation (SD), coefficient of variation (CV), mean, and range—and outcomes, as well as the prognostic role of the stress hyperglycemia ratio (SHR) and admission glucose.

**Methods:**

We conducted a retrospective cohort study using data extracted from the MIMIC-IV database. Blood glucose measurements within the first 48 h of hospital admission were used to calculate glycemic variability metrics and the SHR. Cox proportional hazards regression models, along with restricted cubic spline (RCS) analyses, were employed to evaluate the relationship between these glucose metrics and the primary outcome of 5-year all-cause mortality, as well as the secondary outcome of in-hospital mortality.

**Results:**

A total of 2773 AMI patients were included in the final analysis. Higher glycemic variability, as indicated by increased glucose SD, CV, mean, and range, was significantly associated with elevated mortality risk. In contrast, the associations of SHR and admission glucose with mortality exhibited a U-shaped pattern. Notably, the lowest hazard ratios were observed at an SHR of 0.85 and an admission glucose of 96.3 mg/dL, suggesting that moderate levels of these metrics represent cohort-specific risk minima associated with the most favorable prognosis. Additionally, subgroup analyses revealed that older patients and those with diabetes are particularly vulnerable to adverse outcomes related to extreme glycemic deviations.

**Conclusions:**

Early glycemic variability and stress hyperglycemia are significantly associated with short- and long-term mortality in AMI patients. Moderate levels of SHR and admission glucose correlate with the lowest mortality risk, underscoring the importance of optimizing glycemic stability.

**Supplementary Information:**

The online version contains supplementary material available at 10.1186/s12872-026-05842-5.

## Introduction

Acute myocardial infarction (AMI) continues to be a leading cause of death and disability worldwide, despite significant advances in early diagnosis and treatment strategies [1–4]. The prognosis of AMI patients is influenced by various factors, among which blood glucose levels have emerged as a critical marker of outcomes [5–7]. Hyperglycemia, especially stress-induced hyperglycemia, is commonly observed in AMI patients and has been associated with poor clinical outcomes, including higher mortality and morbidity rates [8–10].

In recent years, the role of blood glucose variability (BGV) has been increasingly acknowledged in the prognosis of critically ill patients. Blood glucose fluctuations, which reflect instability in glucose homeostasis, have been linked to worsened outcomes in a range of diseases, including diabetes and stroke [11–13]. However, the specific relationship between early blood glucose fluctuations and prognosis in AMI patients remains underexplored. Most prior research has predominantly focused on single blood glucose measurements or baseline levels. A critical gap in knowledge persists regarding how dynamic glucose variability during the highly vulnerable first 48 h of intensive care unit (ICU) admission interacts with stress-induced markers to affect both immediate and 5-year survival. What this study adds to the previous literature is a comprehensive evaluation of multiple continuous glycemic metrics alongside the stress hyperglycemia ratio (SHR) and admission glucose. Furthermore, unlike previous studies that assume a linear risk, our work uniquely highlights the non-linear, U-shaped relationship of SHR and admission glucose with mortality in a large critical care cohort, thereby identifying specific optimal glycemic targets to minimize adverse outcomes [14, 15].

This study aims to investigate the relationship between blood glucose fluctuations during the first 48 h of hospitalization and the prognosis of AMI patients, examining both short-term (in-hospital mortality) and long-term (5-year mortality) outcomes. By analyzing multiple blood glucose measurements, as well as additional indicators such as stress hyperglycemia and glycated hemoglobin (HbA1c), we seek to provide a more comprehensive understanding of how these factors, in combination with glucose variability, may influence patient prognosis. Specifically, we will assess the clinical utility of parameters such as mean glucose, standard deviation, range, and HbA1c as prognostic markers, particularly in the context of stress-induced hyperglycemia, to better predict outcomes for AMI patients.

## Materials and methods

### Source of data

The data used in this study were obtained from the Medical Information Mart for Intensive Care IV (MIMIC-IV, version 2.2), a publicly available critical care database developed and maintained by the Massachusetts Institute of Technology (MIT) [16]. This comprehensive database encompasses de-identified electronic health records of patients admitted to the ICUs at the Beth Israel Deaconess Medical Center (BIDMC), a large tertiary academic medical center in Boston, Massachusetts, USA, between the years 2008 and 2019. All data were extracted by the author Ji Jia (Certification No. 43805715), who is authorized to access the database. MIMIC-IV includes detailed clinical information on over 70,000 intensive care unit (ICU) admissions, including demographics, vital signs, laboratory results, medications, and outcome data, making it an ideal resource for studying the relationship between blood glucose fluctuations and prognosis in acute myocardial infarction patients. This project received approval from the Institutional Review Boards of MIT and Beth Israel Deaconess Medical Center (BIDMC), with a waiver of informed consent.

### Selection of participants

Patient data were extracted using Postgres (version 14.5) based on ICD-9/10 codes, identifying individuals diagnosed with AMI, including both acute ST-segment elevation myocardial infarction (STEMI) and non-ST-segment elevation myocardial infarction (NSTEMI). The baseline characteristics considered in this study included age, sex, race, body mass index (BMI), smoking and alcohol use, as well as comorbidities such as hypertension, diabetes, and hyperlipidemia. The treatment regimens evaluated included the use of antiplatelet agents, statins, antihyperglycemic drugs, and insulin therapy. Laboratory data included white blood cell count, platelet count, serum creatinine, HbA1c, admission glucose, and random blood glucose levels. All random blood glucose measurements taken within 48 h of admission were included in the analysis, while for other laboratory measures with multiple values, only the first recorded value was used. All glucose measurements extracted from various sources within the MIMIC-IV database (including core laboratory tests and point-of-care whole blood testing) were harmonized to a standard unit of mg/dL during the data preprocessing phase. Values originally recorded in mmol/L were converted using the standard formula (mmol/L × 18 = mg/dL) to ensure absolute consistency across all analyses.

**Fig. 1:**
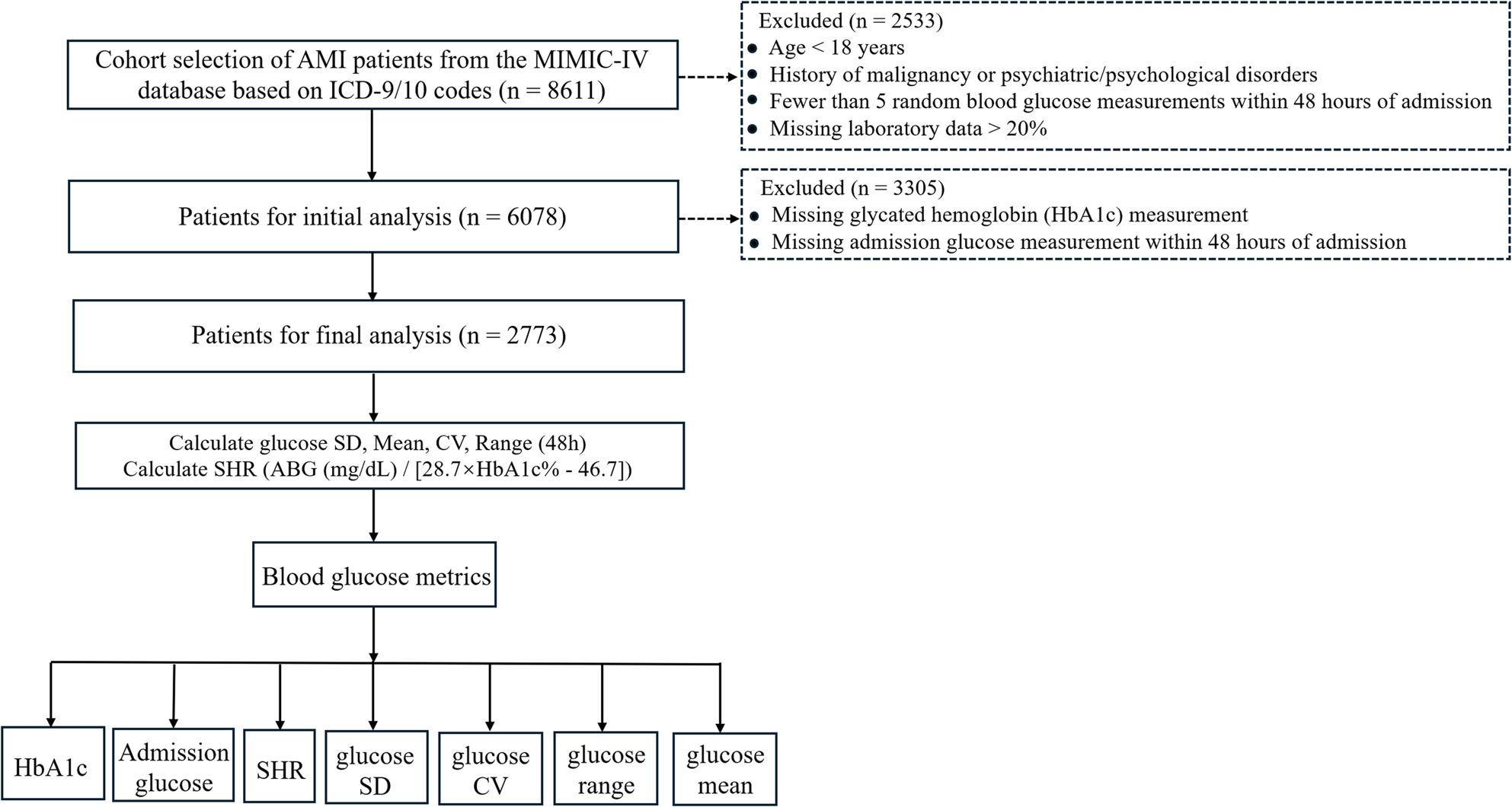
Flowchart of patient selection. SHR: stress hyperglycemia ratio. The terms glucose SD, glucose CV, glucose range, and glucose mean refer to the standard deviation, coefficient of variation, the range (calculated as the maximum value minus the minimum value), and the mean, respectively, of all random blood glucose measurements recorded during the first 48 hours after admission

Patients were excluded from the study if they were under 18 years old, had psychiatric disorders, had fewer than five random blood glucose measurements within the first 48 h of admission, lacked admission glucose data within the first 48 h, had missing HbA1c values, or had missing laboratory data exceeding 20% (Fig. 1). The specific threshold of requiring at least five glucose measurements was established because this minimum data point count is methodologically requisite to calculate reliable and mathematically stable glycemic variability indices (such as SD and CV) that reflect true physiological fluctuations rather than isolated random errors. 

### Calculation of variables

The stress hyperglycemia ratio (SHR) was calculated using the admission glucose and HbA1c values. Admission glucose is explicitly defined as the first random venous plasma glucose measurement obtained upon the patient’s admission to the ICU. The formula applied was: SHR = admission glucose (mg/dL) / (28.7 × HbA1c (%) − 46.7). We used this first admission glucose value consistently for both the SHR calculation and as an independent baseline variable to evaluate acute presentation status. To assess blood glucose variability, several metrics were calculated for all random blood glucose measurements taken within the first 48 h of hospitalization. The standard deviation of random blood glucose levels was calculated as glucose SD, the mean value of these measurements as glucose mean, and the coefficient of variation as glucose CV. Additionally, the range of glucose values was determined by subtracting the minimum value from the maximum value, referred to as glucose range.

### Follow-up and end points

The primary outcome of this study was 5-year all-cause mortality, while the secondary outcome was defined as in-hospital mortality. The time of death events was measured from the point of hospital admission. The median follow-up duration for the study cohort was 1825 days (interquartile range: 1825–1825 days), reflecting the fact that approximately 80% of the patients survived beyond the 5-year observation window and were right-censored at that exact time point. Mortality data were sourced from the Massachusetts State Registry of Vital Records and Statistics. This deterministic linkage between the electronic health records and state-level vital statistics ensures a highly complete ascertainment of both in-hospital and post-discharge mortality.

### Missing data handling

In the MIMIC-IV dataset, missing data for baseline covariates were handled using a rigorous two-step approach to minimize bias. The rates of missingness for relevant continuous variables were all strictly under 20% (detailed missingness rates for all baseline variables are provided in Supplementary Table S3), and no missing values were observed for binary categorical variables. First, to protect the biological integrity of the SHR, we did not impute HbA1c values. Patients lacking baseline HbA1c or admission glucose were directly excluded, ensuring the SHR calculation was based purely on observed clinical data. Second, for the remaining acute glycemic metrics (glucose SD, CV, mean, and range) and other clinical covariates, we utilized Multiple Imputation by Chained Equations (MICE) via the R package ‘mice’ (version 3.16.0). Specifically, we employed the Random Forest imputation algorithm, which robustly handles complex, non-linear relationships in critical care data under the Missing at Random (MAR) assumption, avoiding the variance-shrinkage bias introduced by simple mean or median imputation. Regarding the mortality outcomes, in-hospital mortality was completely documented within the electronic health records. For 5-year all-cause mortality, data were sourced from the Massachusetts State Registry of Vital Records and Statistics up to the database’s available cutoff date. Patients who survived beyond the 5-year observation window, or those whose survival status was unknown at the end of the database’s tracking period, were accounted for using standard right-censoring in the survival analysis. Finally, a complete-case analysis (excluding any patients with missing covariates) was conducted as a sensitivity analysis to confirm the robustness of our imputed models.

### Statistical analysis

Normally distributed continuous variables are presented as mean ± SD and compared using the Student’s t-test. Non-normally distributed continuous variables are presented as median (interquartile range, IQR) and compared using the Wilcoxon rank-sum test. Categorical variables are presented as frequencies and percentages and compared using the chi-squared test. The independent associations between various glycemic parameters and in-hospital as well as five-year all-cause mortality were identified using univariable and multivariable Cox proportional hazards regression models. To avoid severe multicollinearity, each glycemic metric (glucose SD, CV, mean, range, SHR, and admission glucose) was evaluated independently in separate models rather than simultaneously. Collinearity among the baseline clinical covariates within the multivariable models was assessed using the Variance Inflation Factor (VIF), with all VIF values confirming no significant multicollinearity (VIF < 5). We divided six continuous variables, glucose CV, glucose mean, glucose range, glucose SD, SHR, and admission glucose, into four groups based on quartiles, labeled as Q1, Q2, Q3, and Q4, with Q1 serving as the reference group. The median value of each glycemic indicator’s quartile was calculated and entered into a Cox proportional hazards regression model to analyze the relationship between different glycemic indicators and all-cause mortality. Four models were constructed to rigorously evaluate these associations while mitigating potential confounding and overadjustment biases. Model 1 was unadjusted for any covariates. Model 2 adjusted for age, sex, and ethnicity. Model 3 adjusted for age, sex, ethnicity, smoking status, alcohol consumption, hypertension, diabetes, hyperlipidemia, chronic obstructive pulmonary disease (COPD), heart failure, percutaneous coronary intervention (PCI), antiplatelet drugs, and statins. Crucially, to prevent overadjustment and potential collider bias, insulin use was excluded from this primary fully adjusted model. Model 4 served as an additional sensitivity analysis to control for underlying disease acuity and treatment effects; this model adjusted for all variables in Model 3, with the addition of insulin use and the Sequential Organ Failure Assessment (SOFA) score, which was utilized as a robust proxy for baseline illness severity. Hazard ratios (HRs) and their 95% confidence intervals (CIs) and *P*-values were calculated for each group compared to the Q1 reference group. Linear trends were assessed using the likelihood ratio test or Wald test, and trend *P*-values were calculated. Furthermore, restricted cubic spline (RCS) models were utilized to visualize the relationship between various glycemic indices and the risk of 5-year all-cause mortality. The optimal cut-off value for risk stratification was determined based on the Youden index, and Kaplan-Meier (K-M) survival curves with log-rank tests were used to evaluate survival differences between groups. Receiver operating characteristic (ROC) curves were employed in this study to evaluate the predictive performance of various glycemic indices for in-hospital and 5-year all-cause mortality in patients with AMI. All statistical analyses were conducted using R software (version 4.4.2). A two-tailed *P*-value less than 0.05 was used as the threshold for determining statistical significance.

## Results

### Baseline characteristics

A total of 2773 patients with acute myocardial infarction were included in the final study. Among these patients, 1890 (68.2%) were male and 883 (31.8%) were female, with an overall mean age of approximately 68.2 years.

Regarding the secondary outcome of in-hospital mortality, 175 patients died during hospitalization. As shown in Table 1, these patients were significantly older than those who survived to discharge (71.5 vs. 66.5 years, *P* < 0.001). Patients with in-hospital mortality had higher admission glucose and SHR values (*P* < 0.001), although there was no statistically significant difference in baseline HbA1c levels. They also exhibited significantly higher rates of heart failure and COPD, while no statistically significant differences were observed regarding hypertension and diabetes. In terms of clinical management, the in-hospital survival group had a significantly higher proportion of patients undergoing PCI and receiving statins or antiplatelet drugs, whereas the in-hospital mortality group required more frequent insulin therapy (86.3% vs. 67.3%, *P* < 0.001).

**Table 1:** Baseline characteristics of in-hospital survivors and non-survivors

Variables	Total (*n* = 2773)	Survivors (*n* = 2598)	Non-survivors (*n* = 175)	*P*-value
Age, years	68.2 ± 12.7	66.5 ± 12.6	71.5 ± 12.6	<0.001
Sex, %				0.293
Male	1890 (68.2)	1777 (68.4)	113 (64.6)	
Female	883 (31.8)	821 (31.6)	62 (35.4)	
Ethnicity, %				0.007
White	1787 (64.4)	1694 (65.2)	93 (53.1)	
Black	186 (6.7)	174 (6.7)	12 (6.9)	
Hispanic/Latino	81 (2.9)	75 (2.9)	6 (3.4)	
Other	719 (25.9)	655 (25.2)	64 (36.6)	
BMI, kg/m^2^	28.7 [25.3, 32.9]	28.8 [25.3, 33.0]	27.2 [23.8, 32.0]	0.040
Smoking, %	1355 (48.9)	1277 (49.2)	78 (44.6)	0.241
Alcohol use, %	336 (12.1)	305 (11.7)	31 (12.1)	0.019
SOFA	1 [0, 3]	1 [0, 3]	2 [1, 4]	<0.001
Laboratory tests				
Admission glucose, mg/dL	110 [96, 135]	109 [95, 131]	141 [109, 199]	<0.001
HbA1c, %	5.9 [5.6, 6.9]	5.9 [5.6, 6.9]	6.1 [5.6, 6.9]	0.300
SHR	0.88 [0.76, 1.01]	0.88 [0.76, 1.00]	1.10 [0.87, 1.41]	<0.001
HGB, g/L	122.6 ± 21.1	123.1 ± 20.8	115.3 ± 23.4	<0.001
PLT, 10^9^/L	213 [173, 261]	213 [174, 260]	213 [158, 279]	0.500
Scr, umol/L	88.4 [70.7, 114.9]	88.4 [70.7, 114.9]	132.6 [97.2, 185.6]	<0.001
Comorbidity, %				
Hypertension	2111 (76.1)	1969 (75.8)	142 (81.1)	0.108
Hyperlipidemia	1968 (71.0)	1865 (71.8)	103 (58.9)	<0.001
Diabetes	1252 (45.1)	1165 (44.8)	87 (49.7)	0.210
Heart failure	1258 (45.4)	1150 (44.3)	108 (61.7)	<0.001
COPD	438 (15.8)	398 (15.3)	40 (22.9)	0.008
Interventions, %				
Insulin use	1899 (68.5)	1748 (67.3)	151 (86.3)	<0.001
antiplatelet drugs	2734 (98.6)	2569 (98.9)	165 (94.3)	<0.001
Statin	2647 (95.5)	2502 (96.3)	145 (82.9)	<0.001
PCI	1011 (36.5)	968 (37.3)	43 (24.6)	<0.001

Data are expressed as mean ± standard deviation, median [interquartile range] or *n* (%)

*BMI* body mass index, *SHR *stress hyperglycemia ratio, *HGB *hemoglobin, *PLT *platelet, *SCr *serum creatinine, *PCI *percutaneous coronary intervention, *COPD *chronic obstructive pulmonary disease, *SOFA *sequential organ failure assessment score

Regarding the primary outcome, 564 patients experienced all-cause mortality over the 5-year follow-up period. The baseline characteristics stratified by 5-year mortality are presented in Table 2. Similar to the acute phase trends, 5-year non-survivors were significantly older (72.5 vs. 65.4 years, *P* < 0.001), presented with higher SOFA scores, and underwent PCI less frequently (27.3% vs. 38.8%, *P* < 0.001) compared to long-term survivors. Notably, in contrast to the in-hospital cohort, long-term non-survivors demonstrated a significantly higher prevalence of chronic comorbidities, including hypertension (84.8% vs. 73.9%, *P* < 0.001) and diabetes (57.1% vs. 42.1%, *P* < 0.001). Furthermore, alongside elevated admission glucose and SHR, 5-year non-survivors also exhibited significantly higher baseline HbA1c levels (6.1% vs. 5.9%, *P* = 0.001), highlighting the profound impact of chronic glucometabolic dysregulation on long-term prognosis.

**Table 2:** Baseline characteristics of the study population stratified by 5-year all-cause mortality

Variables	Survivors (*n* = 2209)	Non-survivors (*n* = 564)	*P*-value
Age, years	65.4 ± 12.4	72.5 ± 12.2	<0.001
Sex, %			<0.001
Male	1559 (70.6)	331 (58.7)	
Female	650 (29.4)	233(41.3)	
Ethnicity, %			0.031
White	1422 (64.4)	365 (64.7)	
Black	134 (6.1)	52 (9.2)	
Hispanic/Latino	68 (3.1)	13 (2.3)	
Other	585 (26.5)	134 (23.8)	
BMI, kg/m^2^	28.7 [25.4, 32.8]	29.3 [24.8, 33.3]	0.679
Smoking, %	1094 (49.5)	261 (46.3)	0.168
Alcohol use, %	256 (11.6)	80 (14.2)	0.092
SOFA	1 [0, 3]	2 [0, 4]	<0.001
Laboratory tests			
Admission glucose, mg/dL	108 [95, 129]	121 [99, 163]	<0.001
HbA1c, %	5.9 [5.5, 6.8]	6.1 [5.6, 7.1]	0.001
SHR	0.87 [0.76, 0.99]	0.95 [0.76, 1.18]	<0.001
HGB, g/L	126.0 ± 19.7	109.7 ± 21.3	<0.001
PLT, 10^9^/L	213 [175, 259]	210 [159, 276]	0.364
Scr, umol/L	88.4 [70.7, 106.1]	123.8 [79.6, 185.6]	<0.001
Comorbidity, %			
Hypertension	1633 (73.9)	478 (84.8)	<0.001
Hyperlipidemia	1571 (71.1)	397 (70.4)	0.734
Diabetes	930 (42.1)	322 (57.1)	<0.001
Heart failure	860 (38.9)	398 (70.6)	<0.001
COPD	290 (13.1)	148 (26.2)	<0.001
Interventions, %			
Insulin use	1470 (66.5)	429 (76.1)	<0.001
antiplatelet drugs	2187 (99.0)	547 (97.0)	<0.001
Statin	2145 (97.1)	502 (89.0)	<0.001
PCI	857 (38.8)	154 (27.3)	<0.001

Data are expressed as mean ± standard deviation, median [interquartile range] or *n* (%)

*BMI *body mass index, *SHR *stress hyperglycemia ratio, *HGB *hemoglobin, *PLT *platelet, *SCr *serum creatinine, *PCI *percutaneous coronary intervention, *COPD *chronic obstructive pulmonary disease, *SOFA *sequential organ failure assessment score

### Clinical outcomes for all-cause mortality

**Fig. 2:**
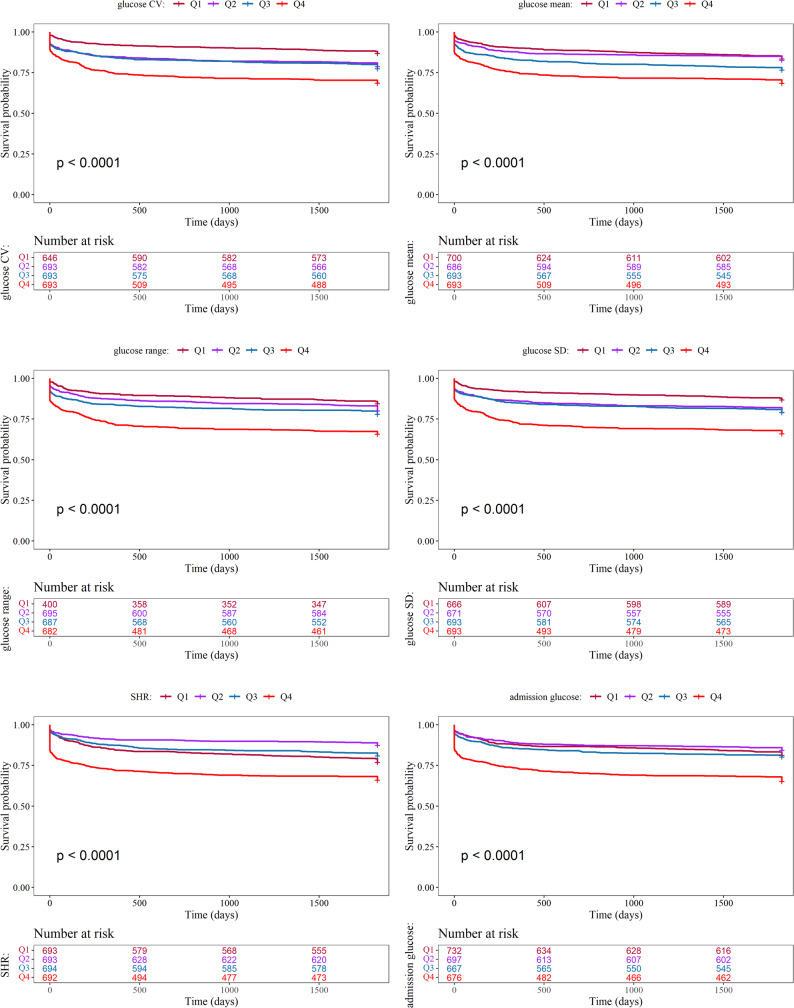
Survival probability analysis of different glucose-related metrics. The terms glucose SD, glucose CV, and glucose mean are used to denote glucose standard deviation, coefficient of variation, and mean, respectively. Glucose range is defined as the maximum glucose value minus the minimum glucose value. Q1–Q4: quartile 1–4

In Fig. 2, the Kaplan-Meier survival curves for the four variables, glucose CV, glucose mean, glucose range, and glucose SD, show that as the quartiles increase from Q1 to Q4, the survival probability gradually decreases, with the highest survival probability in the Q1 group and the lowest in the Q4 group, indicating that increased random blood glucose fluctuation and fluctuation range are associated with poor survival prognosis. For SHR and admission glucose, the survival curves show different trends, with the highest survival probability in the Q2 group and the lowest in the Q4 group, while the survival probabilities of the Q1 and Q3 groups are in the middle. This suggests that moderate levels of SHR and admission glucose are associated with better survival prognosis, while excessively low or high values predict poor prognosis. 

Cox proportional hazards models demonstrated that for glucose SD, glucose CV, glucose mean, and glucose range, the hazard ratios (HRs) significantly increased with increasing quartiles from Q1 to Q4. Notably, across all four models (Models 1 to 4), the Q1 group exhibited the highest survival probability, while the Q4 group had the lowest, with trend *P*-values all less than 0.001. This suggests that elevated standard deviation, coefficient of variation, and range of glucose values are associated with poorer prognosis. For SHR and admission glucose, the pattern differed. The Q2 group had the lowest HRs for both SHR and admission glucose, indicating that moderate levels of these two metrics are associated with lower mortality risk, whereas the Q1 and Q4 groups presented higher risks. Specifically, in the analysis of SHR, for Model 1 without adjustment for any covariates, the HRs for the Q2, Q3, and Q4 groups were 0.52 (95% CI: 0.40–0.68), 0.81 (95% CI: 0.64–1.02), and 1.67 (95% CI: 1.37–2.04), respectively, with a trend *P*-value less than 0.001. In the analysis of admission glucose, for Model 1 without adjustment for any covariates, the HRs for the Q2, Q3, and Q4 groups were 0.82 (95% CI: 0.64–1.06), 1.07 (95% CI: 0.84–1.36), and 2.12 (95% CI: 1.72–2.62), respectively, with a trend *P*-value less than 0.001. This indicates that moderate levels of SHR and admission glucose are associated with better survival prognosis, while excessively low or high values predict poorer outcomes.

In summary, glucose fluctuation-related indicators such as glucose SD, glucose CV, glucose mean, and glucose range are significantly associated with poorer prognosis, whereas SHR and admission glucose exhibit a U-shaped relationship, with moderate levels associated with lower mortality risk. Importantly, these prognostic associations remained robustly significant in our primary fully adjusted model (Model 3), which specifically excluded insulin to avoid collider bias. Furthermore, even after rigorous adjustment for baseline illness severity (SOFA score) and insulin administration in the sensitivity analysis (Model 4), the extreme quartiles of glycemic variability and stress hyperglycemia consistently demonstrated significantly elevated hazard ratios (*P* for trend < 0.001). This comprehensive adjustment strategy confirms that the deleterious impacts of early glucose fluctuations and severe stress hyperglycemia are independent of initial disease acuity and subsequent insulin therapy (Table 3).

**Table 3:** Multivariable Cox regression analyses

	HR (95% CI)				*P* for trend^$^
	Q1	Q2	Q3	Q4	
glucose SD					
Median	2.58	12.02	24.75	55.86	
Model 1	1.00(Ref)	1.66 (1.27, 2.17)^*^	1.68 (1.29, 2.19)^*^	3.01 (2.35, 3.84)^*^	<0.001
Model 2	1.00 (Ref)	1.55 (1.19, 2.03)^*^	1.59 (1.22, 2.08)^*^	2.83 (2.21, 3.62)^*^	<0.001
Model 3	1.00 (Ref)	1.45 (1.11, 1.90)^*^	1.26 (0.96, 1.65)	1.98 (1.53, 2.57)^*^	<0.001
Model 4	1.00 (Ref)	1.46 (1.03, 2.07)^*^	1.29 (0.92, 1.82)	1.94 (1.39, 2.70)^*^	<0.001
glucose CV					
Median	0.02	0.09	0.18	0.33	
Model 1	1.00 (Ref)	1.71 (1.31, 2.24)^*^	1.83 (1.40, 2.38)^*^	2.73 (2.12, 3.51)^*^	<0.001
Model 2	1.00 (Ref)	1.60 (1.22, 2.09)^*^	1.69 (1.30, 2.21)^*^	2.52 (1.96, 3.24)^*^	<0.001
Model 3	1.00 (Ref)	1.44 (1.10, 1.88)^*^	1.32 (1.00, 1.72)^*^	1.79 (1.38, 2.32)^*^	<0.001
Model 4	1.00 (Ref)	1.57 (1.10, 2.23)^*^	1.46 (1.04, 2.06)^*^	1.77 (1.27, 2.47)^*^	<0.001
glucose mean					
Median	98.67	116.50	141.00	200.50	
Model 1	1.00 (Ref)	1.08 (0.83, 1.39)	1.51 (1.19, 1.92)^*^	2.18 (1.74, 2.73)^*^	<0.001
Model 2	1.00 (Ref)	1.08 (0.84, 1.40)	1.49 (1.17, 1.89)^*^	2.35 (1.87, 2.95)^*^	<0.001
Model 3	1.00 (Ref)	1.07 (0.83, 1.39)	1.28 (1.00, 1.64)^*^	1.79 (1.38, 2.32)^*^	<0.001
Model 4	1.00 (Ref)	1.09 (0.78, 1.53)	1.37 (1.00, 1.87)^*^	1.77 (1.27, 2.46)^*^	<0.001
glucose range					
Median	5.00	21.00	52.00	122.50	
Model 1	1.00 (Ref)	1.32 (0.98, 1.78)	1.51 (1.12, 2.02)^*^	2.57 (1.94, 3.40)^*^	<0.001
Model 2	1.00 (Ref)	1.25 (0.92, 1.68)	1.41 (1.05, 1.90)^*^	2.47 (1.86, 3.28)^*^	<0.001
Model 3	1.00 (Ref)	1.15 (0.85, 1.55)	1.14 (0.84, 1.54)	1.80 (1.34, 2.41)^*^	<0.001
Model 4	1.00 (Ref)	1.04 (0.70, 1.54)	1.02 (0.70, 1.50)	1.45 (1.00, 2.11)^*^	<0.001
SHR					
Median	0.67	0.83	0.94	1.16	
Model 1	1.00 (Ref)	0.52 (0.40, 0.68)^*^	0.81 (0.64, 1.02)	1.67 (1.37, 2.04)^*^	<0.001
Model 2	1.00 (Ref)	0.53 (0.41, 0.69)^*^	0.86 (0.68, 1.09)	1.81 (1.48, 2.22)^*^	<0.001
Model 3	1.00 (Ref)	0.64 (0.49, 0.84)^*^	1.03 (0.81, 1.31)	1.83 (1.49, 2.25)^*^	<0.001
Model 4	1.00 (Ref)	0.77 (0.55, 1.08)	1.18 (0.88, 1.57)	2.29 (1.78, 2.95)^*^	<0.001
Admission glucose					
Median	90.00	103.00	120.00	167.00	
Model 1	1.00 (Ref)	0.82 (0.64, 1.06)	1.07 (0.84, 1.36)	2.12 (1.72, 2.62)^*^	<0.001
Model 2	1.00 (Ref)	0.84 (0.66, 1.09)	1.15 (0.90, 1.46)	2.27 (1.84, 2.80)^*^	<0.001
Model 3	1.00 (Ref)	0.87 (0.67, 1.12)	1.18 (0.93, 1.51)	2.04 (1.62, 2.57)^*^	<0.001
Model 4	1.00 (Ref)	0.94 (0.69, 1.29)	1.37 (1.03, 1.84)^*^	2.41 (1.83, 3.19)^*^	<0.001
HbA1c					
Median	5.40	5.80	6.30	8.20	
Model 1	1.00 (Ref)	1.08 (0.84, 1.37)	1.42 (1.14, 1.75)^*^	1.42 (1.14, 1.76)^*^	0.002
Model 2	1.00 (Ref)	1.00 (0.78, 1.28)	1.22 (0.99, 1.51)	1.47 (1.18, 1.83)^*^	<0.001
Model 3	1.00 (Ref)	1.09 (0.85, 1.40)	1.06 (0.83, 1.34)	1.08 (0.82, 1.42)	0.696
Model 4	1.00 (Ref)	1.09 (0.81, 1.47)	1.10 (0.83, 1.45)	0.99 (0.72, 1.36)	0.692

Model 1: Crude model with no adjustment

Model 2: Adjusted for age, sex, and ethnicity

Model 3: Adjusted for age, sex, ethnicity, smoking status, alcohol consumption, hypertension, diabetes, hyperlipidemia, COPD, heart failure, PCI, antiplatelet drugs, and statins

Model 4: Adjusted for age, sex, ethnicity, smoking status, alcohol consumption, hypertension, diabetes, hyperlipidemia, COPD, heart failure, PCI, antiplatelet drugs, statins, insulin use, and SOFA score

*The group exhibited a statistically significant difference when compared with the reference group (Q1)

^$^Test for trend based on variable containing median value for each quartile

### Glucose variability indicators as continuous variables

**Fig. 3:**
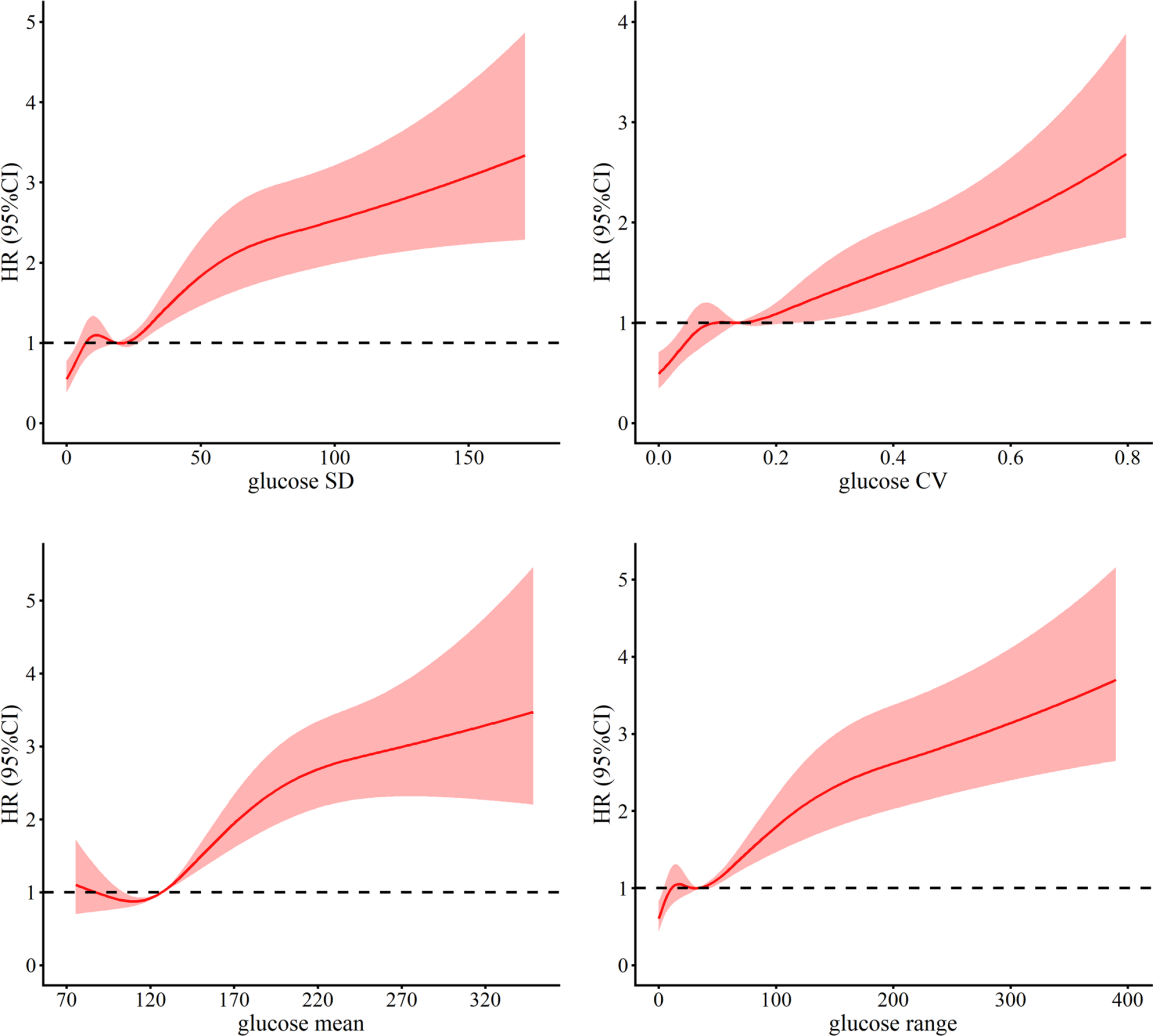
Restricted cubic spline plots illustrate the association of various glucose-related metrics with 5-year all-cause mortality. Hazard ratios are indicated by solid lines and 95% CIs by shaded areas. The horizontal dotted line represents the hazard ratio of 1.0. Analyses were adjusted for age, gender, ethnicity, smoking, alcohol, hypertension, diabetes, hyperlipidemia, COPD, heart failure, PCI, antiplatelet drugs, statin and insulin use

**Fig. 4:**
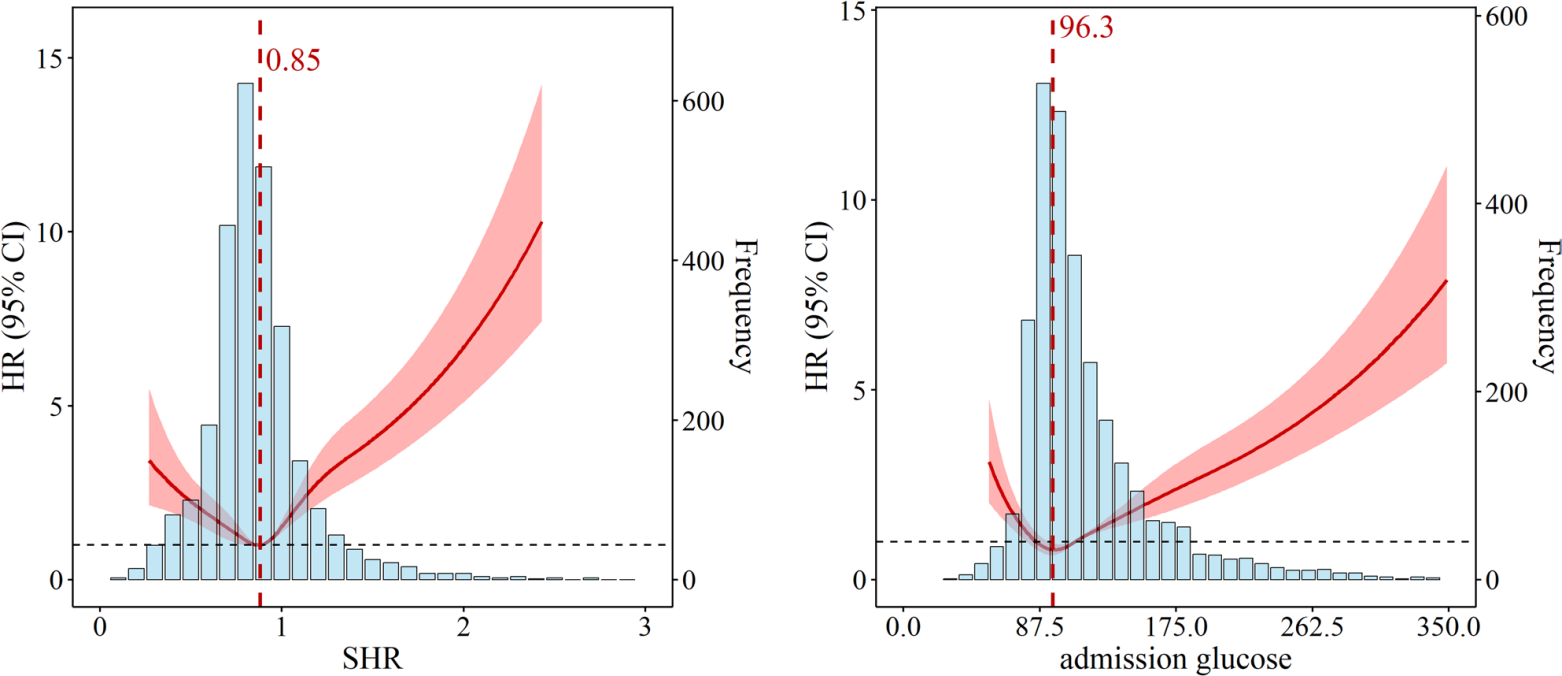
Restricted cubic spline plots illustrate the association of SHR and admission glucose with 5-year all-cause mortality. The distribution of SHR and admission glucose (blue blocks) is presented as a histogram. Hazard ratios are indicated by solid lines, and 95% CIs, by shaded areas. The horizontal dotted line indicates a hazard ratio (HR) of 1.0. Red vertical dotted lines mark the SHR and admission glucose values at the minimum HR. Analyses were adjusted for age, gender, ethnicity, smoking, alcohol consumption, hypertension, diabetes, hyperlipidemia, COPD, heart failure, PCI, antiplatelet drug use, statin use, and insulin use

RCS analysis revealed a non-linear association between glucose variability indices—including glucose SD, glucose CV, glucose range, and glucose mean—and all-cause mortality (Fig. 3). The results indicated that increasing variability and range in glucose measurements were significantly associated with elevated hazard ratios (HRs) for mortality, underscoring the adverse prognostic impact of heightened glycemic fluctuations in patients with AMI. Additionally, RCS curve analysis combined with frequency distribution assessments for the SHR and admission glucose demonstrated a U-shaped relationship with mortality (Fig. 4). This pattern indicated that both excessively low and high levels of SHR and admission glucose were associated with increased mortality risk, whereas intermediate values were linked to a more favorable prognosis. Notably, the lowest HRs were observed at an SHR of approximately 0.85 and an admission glucose level of 96.3 mg/dL, suggesting that these moderate levels may correspond to the most favorable survival outcomes.

**Fig. 5:**
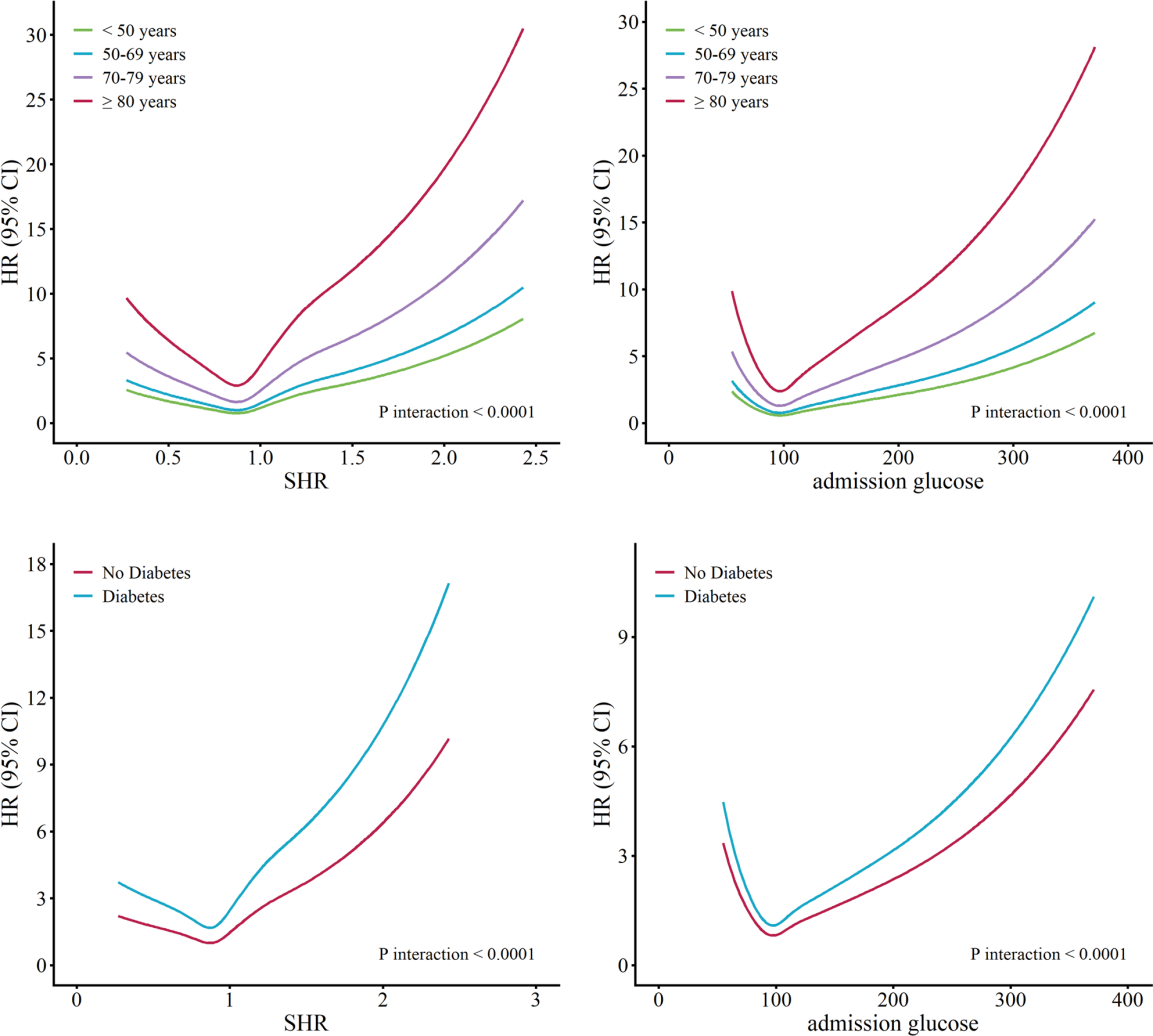
Subgroup analyses using restricted cubic spline plots explored the association of SHR and admission glucose with 5-year all-cause mortality, stratified by age and diabetes status. SHR: stress hyperglycemia ratio, HR = hazard ratio

Further analyses revealed significant effect modification by both age and diabetes status on the prognostic value of key glycemic metrics in patients with AMI. In age-stratified RCS analyses, the relationships between SHR, admission glucose, and mortality exhibited U-shaped or J-shaped patterns among younger patients (< 50 years), with the most protective effects observed at moderate glycemic levels. In contrast, older patients—particularly those aged 80 years or above—demonstrated a steep rise in HRs as SHR and admission glucose levels increased, indicating heightened vulnerability to hyperglycemia in this subgroup. Similarly, stratification by diabetes status revealed that patients without diabetes exhibited a clear protective range at intermediate SHR and admission glucose levels, reflected by lower HRs. Conversely, patients with pre-existing diabetes showed higher baseline mortality risk at lower SHR/ admission glucose values, a less distinct U-shaped association, and a markedly steeper increase in HRs at higher admission glucose concentrations. These findings emphasize the need for individualized glycemic management strategies in AMI patients, with particular attention to older adults and those with diabetes to effectively reduce mortality risk (Fig. 5).

### Discriminative ability of blood glucose metrics for AMI mortality

**Fig. 6:**
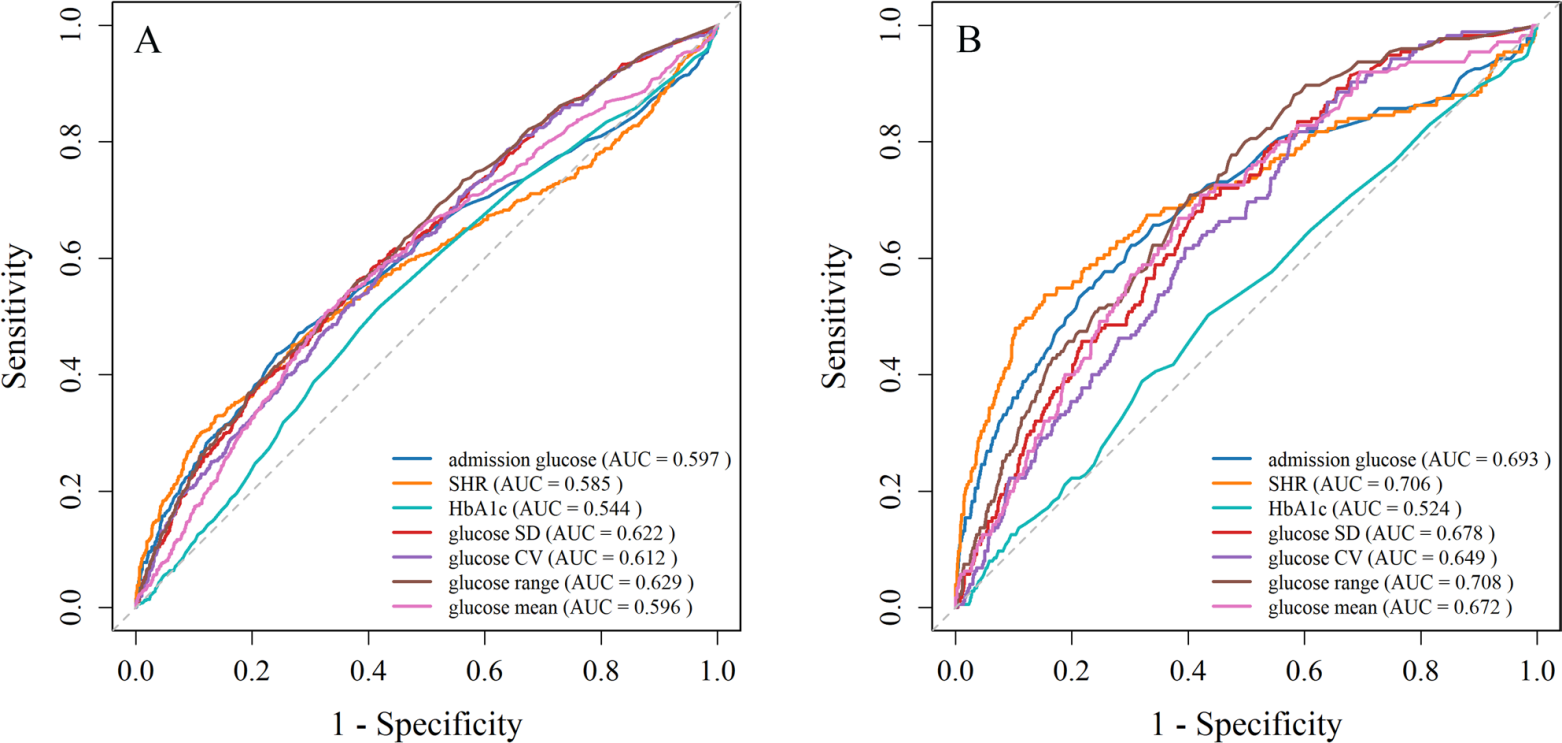
ROC curves illustrate the predictive performance of glucose-related metrics for 5-year (**A**) and in-hospital (**B**) all-cause mortality in patients with myocardial infarction

This study conducted a comprehensive evaluation of ROC curve analyses to explore the discriminative capacity of glucose-related parameters in relation to all-cause mortality in patients with AMI. The analysis demonstrated that glucose range (area under the curve [AUC] = 0.629) and glucose SD (AUC = 0.622) exhibited modest discriminative ability among the isolated glycemic indices, highlighting their potential utility in assessing long-term mortality risk associations. Conversely, HbA1c showed the lowest predictive performance (AUC = 0.544), indicating limited clinical value in forecasting five-year outcomes. These findings support the notion that long-term mortality is influenced by multiple factors, and that glucose variability and range may more effectively reflect chronic metabolic instability than HbA1c, which primarily represents average glycemic control over preceding months. Further confirmation was provided by additional analysis, in which glucose range (AUC = 0.708) and the SHR (AUC = 0.706) demonstrated modest discriminative utility, with admission glucose (AUC = 0.693) performing similarly. Consistent with previous findings, HbA1c yielded the lowest AUC (0.524), underscoring its limited prognostic relevance in the acute setting. The superior predictive values observed for metrics such as SHR and glucose range suggest that these indicators may more accurately capture acute metabolic stress and its immediate implications for in-hospital mortality (Fig. 6).

## Discussion

In this study, we provide robust evidence that early blood glucose fluctuations, quantified by measures such as glucose SD, CV, mean, and range, are strongly associated with both short-term and long-term mortality in AMI patients. These findings not only reiterate the significance of absolute glucose levels but also underscore the prognostic weight carried by glucose stability during the acute phase. Previous research suggests potential mechanisms linking glycemic excursions to adverse outcomes, including increased oxidative stress [17, 18], endothelial dysfunction [19, 20], and inflammation [21, 22]. Importantly, evidence indicates that rapid oscillations in blood glucose trigger a more profound overproduction of reactive oxygen species (ROS) and induce more severe endothelial apoptosis than chronic, sustained hyperglycemia. Furthermore, the steep drops in glucose levels during these fluctuations often trigger compensatory sympathetic nervous system activation. This sympathetic overdrive increases myocardial oxygen demand, exacerbates ischemia, and lowers the threshold for fatal ventricular arrhythmias, thereby offering a robust biological basis for the observed association between heightened glycemic variability and significantly increased mortality risk [23]. Notably, some studies indicate that elevated glycemic variability predicts in-hospital mortality even among AMI patients with normal admission blood glucose levels, further highlighting the importance of monitoring and managing fluctuations beyond single measurements [10].

A particularly notable aspect of our study is the U-shaped relationship observed between the SHR and admission glucose with mortality risk. The RCS analyses revealed that an SHR of 0.85 and an admission glucose of 96.3 mg/dL were associated with the lowest hazard ratios, reflecting a risk nadir where the deleterious effects of both hypoglycemia and hyperglycemia are minimized. While other studies have also reported U-shaped or J-shaped associations between glucose metrics or SHR and outcomes in AMI or coronary artery disease (CAD) patients [24, 25], some have indicated more linear relationships, particularly for SHR [26]. Recent evidence further highlights this complexity; for instance, a recent MIMIC-IV analysis demonstrated that SHR exhibits a U-shaped association with mortality in AMI patients with atrial fibrillation, but a linear relationship in those without it, emphasizing the need to avoid “relative hypoglycemia” in specific subgroups [27]. These discrepancies can likely be explained by differences in study populations and the timing of measurements. Studies reporting linear relationships often involve unselected, less severe cohorts where the primary risk is driven by undiagnosed or poorly controlled chronic diabetes. In contrast, our study focuses exclusively on critically ill AMI patients in the ICU. In this highly vulnerable population, severe acute physiological stress not only induces extreme hyperglycemia but also exhausts physiological reserves, making patients highly susceptible to severe hypoglycemia (spontaneous or iatrogenic). This distinct dual risk in critical care settings effectively drives the U-shaped prognostic curve observed in our study. The specific SHR and admission glucose minima identified in our study reflect cohort-specific baseline values associated with the lowest observed risk, highlighting a specific risk nadir. However, due to the observational design and potential residual confounding, these values indicate prognostic associations rather than interventional treatment targets. This U-shaped pattern highlights the complexities of glycemic control: clinicians must avoid not only the direct tissue injury, osmotic diuresis, and prothrombotic states associated with hyperglycemia but also the potential harm from hypoglycemia (or relative hypoglycemia in diabetics) [28], which can trigger counter-regulatory hormonal responses and increase arrhythmogenic risk [29, 30]. Furthermore, our finding that SHR demonstrated robust predictive performance, potentially superior to admission glucose or HbA1c alone, aligns with the concept that SHR better reflects acute stress-induced hyperglycemia relative to the patient’s chronic glycemic background, thereby offering a more refined prognostic assessment. This concept is strongly supported by newly published cohorts demonstrating that SHR is a formidable independent predictor of specific post-infarction complications, such as in-hospital heart failure following anterior STEMI [31] and type 4a myocardial infarction in NSTEMI patients undergoing PCI, outperforming absolute glucose or HbA1c levels [32]. Nevertheless, it is important to acknowledge that baseline HbA1c remains a valuable independent predictor for major adverse cardiovascular events in specific high-risk subpopulations, such as diabetic patients following revascularization [33].

Stratified analyses further elucidate the complexity of glycemic control in the context of patient heterogeneity. Our data indicate that older patients, particularly those aged 70 years and above, exhibit a markedly steeper increase in mortality risk with deviations from the risk nadir. This age-related vulnerability may be attributed to diminished physiological reserves and an increased propensity for comorbidities that exacerbate the metabolic stress associated with AMI. Similarly, the differential patterns observed between patients with and without diabetes underscore the need for tailored glycemic management strategies [34]. While non-diabetic individuals demonstrated a clear protective effect at moderate glycemic levels, patients with pre-existing diabetes showed higher baseline risks and a blunted protective response, especially exhibiting markedly increased hazard ratios at higher admission glucose concentrations. This may reflect how chronic metabolic dysregulation impairs adaptive responses to acute glycemic fluctuations, possibly involving underlying endothelial dysfunction or altered counter-regulatory mechanisms [35].

The clinical implications of these findings are significant. They suggest that therapeutic interventions in AMI should not solely focus on rapid normalization of blood glucose but rather aim to achieve and maintain glycemic stability. Operating near this risk nadir presents considerable challenges, requiring strategies that minimize both hyperglycemia and the risk of iatrogenic hypoglycemia, which is itself associated with adverse outcomes [36]. This may necessitate more sophisticated management approaches, potentially including continuous glucose monitoring (CGM) to better detect variability, alongside judicious insulin protocols. Furthermore, incorporating glycemic variability metrics and SHR into existing risk stratification models could enhance predictive accuracy for adverse outcomes in this high-risk population [5, 37].

Moving forward, future research should focus on several promising directions to build upon these findings. First, prospective, multicenter randomized controlled trials are needed to validate whether protocolized glycemic control targeting these specific risk nadirs can actively reduce mortality in AMI patients. Second, the integration of continuous glucose monitoring (CGM) technologies in the ICU setting should be investigated to determine if real-time tracking of glucose variability can prevent silent hypoglycemic episodes and improve outcomes. Finally, incorporating dynamic glycemic variability metrics into machine-learning-based prognostic models could further refine individualized risk stratification and clinical decision-making for critically ill cardiac patients.

### Limitations of the study

This study has several limitations that warrant consideration. First, the retrospective design inherently limits the ability to establish causal relationships between glycemic variability and mortality outcomes. The use of data from the MIMIC-IV database, while comprehensive, may introduce selection bias and limit the generalizability of our findings to other populations or healthcare settings. Second, although we adjusted for multiple confounding factors, residual confounding cannot be entirely excluded, particularly given the complexity of metabolic and cardiovascular interactions in AMI patients. Third, the frequency and timing of blood glucose measurements varied among patients, which could affect the precision of calculated variability metrics such as glucose SD, CV, mean, and range. Furthermore, the requirement of at least five glucose measurements within the first 48 h introduces a specific selection bias. As detailed in Supplementary Table S2, compared to excluded patients, the included cohort received more intensive acute management (e.g., significantly higher rates of insulin use and PCI). Conversely, excluded patients were generally older, had a higher prevalence of heart failure, and exhibited higher in-hospital mortality, which likely reflects early deaths occurring before sufficient glucose measurements could be recorded. Consequently, our findings are most applicable to AMI patients who survive the hyperacute phase and undergo intensive ICU monitoring and management, and should be extrapolated with caution to either rapidly fatal cases or stable ward patients without frequent glucose monitoring. Additionally, the lack of data on certain clinical interventions and patient-specific factors that might influence glycemic control (e.g., nutritional support, concurrent medications, and insulin administration protocols) further limits the interpretation of our results. Finally, the reliance on a single-center dataset (from a tertiary academic medical center) fundamentally shapes the clinical profile of our cohort. For instance, the rate of PCI in our study population was 36.5%, which is notably lower than general cardiology registries. This highlights a specific characteristic of the MIMIC-IV database: it exclusively captures critically ill patients admitted to the ICU. Uncomplicated AMI patients who undergo successful primary PCI are often admitted to standard cardiac wards and thus bypass this database. Consequently, our cohort is inherently enriched with highly complex patients who may possess contraindications to invasive procedures due to severe multi-organ dysfunction, frailty, or advanced age, leading to higher rates of conservative management or alternative strategies like coronary artery bypass grafting (CABG). This unique ICU demographic profile, despite its large size, may restrict the external validity of our conclusions when generalized to typical, uncomplicated AMI populations, highlighting the need for prospective, multicenter studies to confirm these observations. Furthermore, although we utilized robust multiple imputation methods for variables with missing data, the process of imputation inherently introduces some degree of statistical uncertainty compared to fully observed datasets. Additionally, the use of standard ROC curves to evaluate the discriminative ability for 5-year mortality is limited, as this conventional approach does not account for the censoring inherent in time-to-event data. Finally, as a retrospective analysis of an existing database, this study was not pre-registered on a clinical trial platform, which prevents the prospective evaluation of our analytical protocols.

## Conclusion

Our study demonstrates that early glycemic variability and stress hyperglycemia play critical roles in predicting both short-term and long-term mortality in acute myocardial infarction patients. Notably, while higher variability metrics are associated with increased mortality risk, the U-shaped relationship observed for the stress hyperglycemia ratio and admission glucose indicates that moderate levels (specifically, an SHR of 0.85 and an admission glucose of 96.3 mg/dL) are linked to the most favorable outcomes. These findings emphasize the importance of not only controlling absolute glucose levels but also minimizing fluctuations during the acute phase of AMI. Tailored glycemic management strategies, particularly for older patients and those with diabetes, may be essential to improve survival and long-term prognosis. Future prospective studies are warranted to validate these findings and to explore the underlying mechanisms driving the observed associations.

## Supplementary Information

Supplementary Material 1.

Supplementary Material 2.

Supplementary Material 3.

## Data Availability

The datasets utilized in this study are available in publicly accessible online repositories. Detailed information regarding the repositories and their respective accession numbers can be found as follows: MIMIC-IV repository (https://physionet.org/content/mimiciv/2.2/).

## Abbreviations

MIMIC: Medical Information Mart for Intensive Care
MIT: Massachusetts Institute of Technology
BIDMC: Beth Israel Deaconess Medical Center
ICU: Intensive care unit
AMI: Acute myocardial infarction
STEMI: ST-segment elevation myocardial infarction
NSTEMI: Non-ST-segment elevation myocardial infarction
SD: Standard deviation
CV: Coefficient of variation
SHR: Stress hyperglycemia ratio
BGV: Blood glucose variability
HbA1c: Glycated hemoglobin
RCS: Restricted cubic spline
COPD: Chronic obstructive pulmonary disease
PCI: Percutaneous coronary intervention
BMI: Body mass index
HGB: Hemoglobin
PLT: Platelet
SCr: Serum creatinine
ICD-9/10: International Classification of Diseases, Ninth/Tenth Revision
AUC: Area under curve
ROC: The receiver operating characteristic curves
HRs: Hazard ratios
CIs: Confidence intervals
