# Supplementary material for: Association of early blood glucose metrics with short- and long-term prognosis in acute myocardial infarction patients: a retrospective cohort study
Source: BMC Cardiovasc Disord. 2026 Apr 8;26:425. doi: 10.1186/s12872-026-05842-5 (PMC13196221; doi:10.1186/s12872-026-05842-5)
Supplement: Supplementary file 1 — Supplementary Material 1. [file 12872_2026_5842_MOESM1_ESM.docx]

Table S1 Complete-case sensitivity analysis for the association between early glycemic metrics and 5-year all-cause mortality (n = 1913)

|  | | HR (95% CI) | | | | | *P* for trend^$^ | |
| --- | --- | --- | --- | --- | --- | --- | --- | --- |
|  |  | Q1 | Q2 | Q3 | | Q4 |  |  |
| glucose SD | |  |  |  | |  |  | |
| Median | | 2.58 | 12.02 | 24.75 | | 55.86 |  | |
| Model 3  Model 4 | | 1.00 (Ref)  1.00 (Ref) | 1.42 (1.00, 2.01)^*^  1.46 (1.03, 2.07)^*^ | | 1.25 (0.89, 1.77)  1.29 (0.92, 1.82) | 1.88 (1.35, 2.62)^*^  1.94 (1.39, 2.70)^*^ | ＜0.001  ＜0.001 | |
| glucose CV | |  |  |  | |  |  | |
| Median | | 0.02 | 0.09 | 0.18 | | 0.33 |  | |
| Model 3 | 1.00 (Ref) | | 1.53 (1.08, 2.16)^*^ | | 1.43 (1.01, 2.01)^*^ | 1.74 (1.25, 2.43)^*^ | | 0.005 |
| Model 4 | | 1.00 (Ref) | 1.57 (1.11, 2.23)^*^ | | 1.46 (1.04, 2.06)^*^ | 1.77 (1.27, 2.47)^*^ | | 0.004 |
| glucose mean | |  |  |  | |  |  | |
| Median | | 98.67 | 116.50 | 141.00 | | 200.50 |  | |
| Model 3 | 1.00 (Ref) | | 1.07 (0.77, 1.50) | | 1.35 (0.98, 1.84) | 1.70 (1.22, 2.36)^*^ | ＜0.001 | |
| Model 4 | | 1.00 (Ref) | 1.09 (0.78, 1.53) | | 1.37 (1.00, 1.87)^*^ | 1.77 (1.27, 2.46)^*^ | ＜0.001 | |
| glucose range | |  |  |  | |  |  | |
| Median | | 5.00 | 21.00 | 52.00 | | 122.50 |  | |
| Model 3 | | 1.00 (Ref) | 1.03 (0.70, 1.52) | | 1.00 (0.68, 1.47) | 1.43 (0.99, 2.08) | | 0.002 |
| Model 4 | | 1.00 (Ref) | 1.04 (0.70, 1.54) | | 1.02 (0.70, 1.50) | 1.45 (1.00, 2.11)^*^ | | 0.002 |
| SHR | |  |  |  | |  |  | |
| Median | | 0.67 | 0.83 | 0.94 | | 1.16 |  | |
| Model 3 | | 1.00 (Ref) | 0.75 (0.54, 1.06) | 1.17 (0.87, 1.56) | | 2.27 (1.77, 2.92)^*^ | ＜0.001 | |
| Model 4 | | 1.00 (Ref) | 0.77 (0.55, 1.08) | | 1.18 (0.88, 1.57) | 2.29 (1.78, 2.95)^*^ | ＜0.001 | |
| VBG | |  |  |  | |  |  | |
| Median | | 90.00 | 103.00 | 120.00 | | 167.00 |  | |
| Model 3 | | 1.00 (Ref) | 0.93 (0.69, 1.28) | | 1.36 (1.02, 1.82)^*^ | 2.39 (1.81, 3.15)^*^ | ＜0.001 | |
| Model 4 | | 1.00 (Ref) | 0.94 (0.69, 1.29) | | 1.37 (1.03, 1.84)^*^ | 2.41 (1.83, 3.19)^*^ | ＜0.001 | |
| HbA1c | |  |  |  | |  |  | |
| Median | | 5.40 | 5.80 | 6.30 | | 8.20 |  | |
| Model 3 | | 1.00 (Ref) | 1.08 (0.80, 1.46) | | 1.10 (0.83, 1.45) | 0.96 (0.70, 1.32) | 0.573 | |
| Model 4 | | 1.00 (Ref) | 1.09 (0.81, 1.47) | | 1.10 (0.83, 1.45) | 0.99 (0.72, 1.36) | | 0.692 |

Model 3: Adjusted for age, sex, ethnicity, smoking status, alcohol consumption, hypertension, diabetes, hyperlipidemia, COPD, heart failure, PCI, antiplatelet drugs, and statins. Model 4: Adjusted for age, sex, ethnicity, smoking status, alcohol consumption, hypertension, diabetes, hyperlipidemia, COPD, heart failure, PCI, antiplatelet drugs, statins, insulin use, and SOFA score. *The group exhibited a statistically significant difference when compared with the reference group (Q1). $Test for trend based on variable containing median value for each quartile. This complete-case sensitivity analysis exclusively utilized patients with no missing data across all included covariates to validate the robustness of the primary imputed models.
