# Supplementary material for: Association of early blood glucose metrics with short- and long-term prognosis in acute myocardial infarction patients: a retrospective cohort study
Source: BMC Cardiovasc Disord. 2026 Apr 8;26:425. doi: 10.1186/s12872-026-05842-5 (PMC13196221; doi:10.1186/s12872-026-05842-5)
Supplement: Supplementary file 2 — Supplementary Material 2. [file 12872_2026_5842_MOESM2_ESM.docx]

Table S2 Baseline characteristics of included and excluded patients.

| Variables | | Total  (n = 8611) | | Included  (n = 2773) | | Excluded  (n = 5838) | *P*-value |
| --- | --- | --- | --- | --- | --- | --- | --- |
| Age, years | | 69.1 ± 13.5 | | 66.8 ± 12.7 | | 70.2 ± 13.7 | ＜0.001 |
| Sex, % | |  | |  | |  | ＜0.001 |
| Male | | 5340 (62.0) | | 1890 (68.2) | | 3450 (59.1) |  |
| Female | | 3271 (38.0) | | 883 (31.8) | | 2388 (40.9) |  |
| Ethnicity, % | |  | |  | |  | ＜0.001 |
| White | | 5942 (69.0) | | 1787 (64.4) | | 4155 (71.2) |  |
| Black | | 742 (8.6) | | 186 (6.7) | | 556 (9.5) |  |
| Hispanic/Latino | | 226 (2.6) | | 81 (2.9) | | 145 (2.5) |  |
| Other | | 1701 (19.8) | | 719 (25.9) | | 982 (16.8) |  |
| BMI, kg/m^2^ | | 28.2 [24.8, 32.5] | | 28.7 [25.2, 32.9] | | 27.9 [24.4, 32.1] | ＜0.001 |
| Smoking, % | | 3852 (44.7) | | 1355 (48.9) | | 2497 (42.8) | ＜0.001 |
| Alcohol use, % | | 1090 (12.7) | | 336 (12.1) | | 754 (12.9) | 0.298 |
| SOFA | | 1 [0, 3] | | 1 [0, 3] | | 1 [0, 3] | ＜0.010 |
| In-hospital mortality, % | | 918 (10.7) | | 175 (6.3) | | 743 (12.7) | ＜0.010 |
| Laboratory tests | | | | | | | |
| VBG, mg/dL | | 108 [94, 137] | | 110 [96, 135] | | 107 [94, 139] | 0.047 |
| HbA1c, % | | 5.9 [5.6, 6.9] | | 5.9 [5.6, 6.9] | | 5.4 [5.2, 5.5] | 0.135 |
| SHR | | 0.88 [0.76, 1.01] | | 0.88 [0.76, 1.01] | | 1.05 [0.98, 1.12] | 0.274 |
| HGB, g/L | | 115.5 ± 22.6 | | 122.6 ± 21.1 | | 112.0 ± 22.5 | ＜0.001 |
| PLT, 10^9^/L | | 212 [166, 266] | | 213 [173, 261] | | 211 [163, 268] | 0.110 |
| Scr, umol/L | 97.2 [70.7, 132.6] | | 88.4 [70.7, 114.9] | | 97.2 [70.7, 150.3] | | ＜0.001 |
| Comorbidity, % | | | | | | | |
| Hypertension | | 6638 (77.1) | | 2111 (76.1) | | 4527 (77.5) | 0.144 |
| Hyperlipidemia | | 6248 (72.6) | | 1968 (71.0) | | 4280 (73.3) | 0.023 |
| Diabetes | | 3714 (43.1) | | 1252 (45.1) | | 2462 (42.2) | 0.009 |
| Heart failure | | 4570 (53.1) | | 1258 (45.4) | | 3312 (56.7) | ＜0.001 |
| COPD | | 1679 (19.5) | | 438 (15.8) | | 1241 (21.3) | ＜0.001 |
| Interventions, % | | | | | | | |
| Insulin use | | 4570 (53.1) | | 1899 (68.5) | | 2671 (45.8) | ＜0.001 |
| antiplatelet drugs | | 8130 (94.4) | | 2734 (98.6) | | 5396 (92.4) | ＜0.001 |
| Statin | | 7733 (89.8) | | 2647 (95.5) | | 5086 (87.1) | ＜0.001 |
| PCI | | 2264 (26.3) | | 1011 (36.5) | | 1253 (21.5) | ＜0.001 |

Data are expressed as mean ± standard deviation, median [interquartile range] or n (%). BMI: body mass index; SHR: stress hyperglycemia ratio; VBG: venous blood glucose; HGB: hemoglobin; PLT: platelet; SCr: serum creatinine; PCI: percutaneous coronary intervention; COPD: chronic obstructive pulmonary disease. SOFA: sequential organ failure assessment score.
