# Supplementary material for: Association of early blood glucose metrics with short- and long-term prognosis in acute myocardial infarction patients: a retrospective cohort study
Source: BMC Cardiovasc Disord. 2026 Apr 8;26:425. doi: 10.1186/s12872-026-05842-5 (PMC13196221; doi:10.1186/s12872-026-05842-5)
Supplement: Supplementary file 3 — Supplementary Material 3. [file 12872_2026_5842_MOESM3_ESM.docx]

Table S3. The proportion of missing data for variables included in the analysis

| Variable | Missing Count (n) | | Missing Rate (%) | |
| --- | --- | --- | --- | --- |
| Age | | 0 | | 0 |
| gender | | 0 | | 0 |
| Ethnicity | | 0 | | 0 |
| Smoking | | 0 | | 0 |
| Alcohol use | | 0 | | 0 |
| BMI | | 349 | | 12.6 |
| SOFA | | 497 | | 18.2 |
| VBG | | 0 | | 0 |
| HbA1c | | 0 | | 0 |
| HGB | | 129 | | 4.7 |
| PLT | | 33 | | 1.2 |
| Scr | | 3 | | 0.1 |
| Hypertension | | 0 | | 0 |
| Hyperlipidemia | | 0 | | 0 |
| Diabetes | | 0 | | 0 |
| Heart failure | | 0 | | 0 |
| COPD | | 0 | | 0 |
| Insulin use | | 0 | | 0 |
| antiplatelet drugs | | 0 | | 0 |
| Statin | | 0 | | 0 |
| PCI | | 0 | | 0 |

BMI: body mass index; VBG: venous blood glucose; HGB: hemoglobin; PLT: platelet; SCr: serum creatinine; PCI: percutaneous coronary intervention; COPD: chronic obstructive pulmonary disease. SOFA: sequential organ failure assessment score.
